# Thyroid arterial embolization in a patient with congenital heart disease and refractory amiodarone-induced thyrotoxicosis

**DOI:** 10.1530/ETJ-21-0007

**Published:** 2021-09-02

**Authors:** Bruno Bouça, Ana Cláudia Martins, Paula Bogalho, Lídia Sousa, Tiago Bilhim, Filipe Veloso Gomes, Élia Coimbra, Ana Agapito, José Silva-Nunes

**Affiliations:** 1 Endocrinology, Diabetes and Metabolism Department, Centro Hospitalar Universitário Lisboa Central, Lisbon, Portugal; 2 Cardiology Department, Centro Hospitalar Universitário Lisboa Central, Lisbon, Portugal; 3 Interventional Radiology Department, Centro Hospitalar Universitário Lisboa Central, Lisbon, Portugal

**Keywords:** amiodarone, thyrotoxicosis, embolization, congenital heart disease

## Abstract

**Introduction:**

Amiodarone-induced thyrotoxicosis (AIT) can sometimes lead to life-threatening complications, especially in patients with congenital heart disease (CHD). We report the case of a patient with refractory AIT that was successfully treated with thyroid arterial embolization (TAE).

**Case report:**

A 34-year-old man with complex cyanotic CHD complicated with heart failure (HF), pulmonary hypertension, and supraventricular tachyarrhythmias, was treated with amiodarone since 2013. In March 2019, he presented worsening of his cardiac condition and symptoms of thyrotoxicosis that were confirmed by laboratory assessment. Thiamazole 30 mg/day and prednisolone 40 mg/day were prescribed, but the patient experienced worsening of his cardiac condition with several hospital admissions in the next 5 months, albeit increasing dosages of thionamide and glucocorticoid and introduction of cholestyramine and lithium. Thyroidectomy was excluded due to the severity of thyrotoxicosis, and plasmapheresis was contraindicated due to the cardiac condition. TAE of the four thyroid arteries was then performed with no immediate complications. Progressive clinical and analytical improvement ensued with gradual reduction and suspension of medication with the patient returning to euthyroid state and his usual cardiac condition previous to the AIT.

**Conclusion:**

For patients with medication refractoriness and whose condition precludes thyroidectomy, embolization of thyroid arteries may be an effective and safe option.

**Established facts:**

- Amiodarone-induced thyrotoxicosis (AIT) can be refractory to a combination therapy of thionamides and glucocorticoids.
- Restoration of euthyroidism is of paramount importance in heart failure (HF) patients.
- Emergency thyroidectomy for AIT unresponsive to medical therapy is recommended in patients with severe underlying cardiac disease or deteriorating cardiac function.

**Novel insights:**

- Thyroid arterial embolization (TAE) appeared as a salvage therapy in this patient.
- To the best of our knowledge, few case reports in the literature have described the embolization of the four thyroid arteries in AIT context.
- Endovascular embolization techniques are a valuable therapeutic option and can be considered in cases where standard forms of treatment are ineffective or involve unacceptable risks.

## Introduction

The number of patients with congenital heart disease (CHD) reaching adulthood has largely increased due to advances in percutaneous and surgical techniques. Repaired and unrepaired CHD is commonly associated with atrial and ventricular tachyarrhythmias. The more complicated the congenital heart defect and the palliation required, the higher and earlier the incidence of atrial arrhythmias (1). These are associated with thromboembolic phenomena and hemodynamic compromise and are, thus, a major cause of morbidity and mortality. Amiodarone is widely considered to be the most effective antiarrhythmic drug, even for the CHD population. Treatment with this class III anti-arrhythmic drug is commonly used in these patients as it is very effective for both supra- and ventricular tachyarrhythmia, is hemodynamically well tolerated by patients with impaired inotropic function and/or pre-existent hypotension, and also prevents sudden cardiac death (2).

Due to its high iodine content (37.5% of its molecular weight) and structural similarity to thyroxine, abnormalities in thyroid function are common, especially with long-term use. The reported incidence of thyrotoxicosis is 3–9% in the general population, increasing to 21% in adults with CHD (3, 4). Two main forms of amiodarone-induced thyrotoxicosis (AIT) can occur: type 1 AIT is an iodine-induced hyperthyroidism in nodular goiter or in latent Graves’ disease; type 2 AIT is an amiodarone-induced destructive thyroiditis occurring in a normal thyroid gland. A mixed type is also recognized. AIT can be a dangerous condition because it may exacerbate underlying cardiac abnormalities leading to increased morbidity and mortality, especially in patients with left ventricular dysfunction (5). Restoration of euthyroidism is of paramount importance in heart failure (HF) patients; it may be difficult to achieve with medication and it can also be refractory to a combination therapy of thionamides and glucocorticoids, making this a challenging scenario (2).

The European Thyroid Association recommends emergency thyroidectomy for AIT unresponsive to medical therapy in patients with severe underlying cardiac disease or deteriorating cardiac function (6). Nevertheless, this decision must be made by a multidisciplinary team assessing the balanced risk-benefit in the individual patient. Thyroid arterial embolization (TAE) has been successfully used in toxic goiter, recurrent goiter, Graves’ disease, and thyroid carcinoma. However, there are few reported cases of TAE in the context of AIT (7).

We describe the case of a male patient with complex cyanotic CHD, who developed AIT refractory to medical therapy that was controlled with TAE.

## Case report

A 34-year-old Caucasian male, born with a complex cyanotic CHD (D-transposition of the great arteries and interventricular communication), was submitted to palliative cardiac surgery (Senning procedure) when he was 2 years old. HF and pulmonary hypertension were early recognized during the course of the disease. In 2010, the patient had an episode of severe hemoptysis with arterial embolization of the collateral bronchial arteries. In 2013, due to supraventricular tachyarrhythmias (atrial fibrillation and flutter), treatment with amiodarone was initiated. In March 2019, he presented to his cardiologist with accentuation of fatigue and cyanosis, weight loss, irritability, and tremor of the extremities that had started in the previous 3 months. Laboratory studies showed Hb 22.1 g/dL, Hct 63.6%, TSH 0.02 uIU/mL (0.35–4.94), FT4 2.12 ng/dL (0.70–1.48), FT3 5.86 pg/mL (1.88–3.18). The patient was referred to our Endocrinology Department in April 2019. On physical examination, the patient’s blood pressure was 100/60 mmHg with an arrythmic pulse of 78 bpm and a respiratory rate of 18 cycles/min. BMI was 20.8 kg/m^2^ (weight 63.7 kg; height 1.75 m). Skin and mucous membranes were severely cyanosized and flushed, with no signs of ophthalmopathy. A normal sized and fibroelastic thyroid gland was palpable, with no nodules.

Analytic reassessment showed TSH <0.01 mIU/L, FT4 4.60 ng/dL, FT3 14.57 pg/mL, and TSH-receptor antibody (TRAb), anti-thyroperoxidase and anti-thyroglobulin were negative. Thyroid ultrasound showed a mobile and well-defined gland, with slightly increased dimensions: right lobe 55 × 20 × 27 mm; left lobe 52 × 18 × 24 mm (longitudinal/transverse/anteroposterior diameters); isthmus (9 mm) diffusely hypoechoic, heterogeneous and hypovascular, with no nodules or adenomegaly. Thyroid scintigraphy was not performed. He started treatment with thiamazole 30mg/day and prednisolone 20 mg/day.

On the 11th day of treatment, the patient had worsening of his cardiac condition with ventricular tachyarrhythmia and syncope, requiring hospitalization in the intensive care unit (ICU) for 17 days and an implantable cardioverter-defibrillator placement. During this period, amiodarone was replaced by dofetilide, bisoprolol was initiated (2.5 mg twice daily), and thiamazole and prednisolone dosages were increased (45 mg and 40 mg daily, respectively). On discharge, there was a substantial clinical and biochemical improvement: TSH <0.01 mIU/L, FT4 2.25 ng/dL and FT3 6.23 pg/mL.

On the 50th day of treatment, despite some clinical improvement, the laboratory check showed an increase in FT4 and FT3 (FT4 2.37 ng/dL and FT3 7.28 pg/mL, respectively) leading to a further increase in thiamazole dosage (60 mg/day).

On the 72nd day, he presented worsening cyanosis and tiredness, palpitations, and peripheral tremor as well as cushingoid facies; the analytical results showed TSH <0.01 mIU/L, FT4 3.95 ng/dL and FT3 12.19 pg/mL. There was some concern regarding patient treatment compliance as he admitted skipping some of the cardiology pills. Therefore, it was decided to hospitalize the patient to stabilize his clinical condition and ensure proper medication intake, increasing daily medical therapy dosages (thiamazole 75 mg and dexamethasone 10 mg) and introducing cholestyramine 16 g/day and lithium 400 mg/day, as adjunctive therapies. Given the clinical and biochemical improvement (TSH <0.01 mIU/L, FT4 3.54 ng/dL and FT3 6.97 pg/mL), the patient was discharged 3 weeks later, on thiamazole 60 mg, prednisolone 60 mg, cholestyramine 16 g and lithium 400 mg daily.

For the following 15 days, he maintained his usual condition in NYHA class II-III. However, on the 119th day, the patient experienced abrupt worsening of cardiac condition, being unable to carry out any physical activity without discomfort and presenting with polypnea, severe bilateral peripheral edema, jugular vein distension, and weight gain (63.4 kg to 69.1 kg). At this point, patient corroborated that all drugs were taken except for cholestyramine. He was admitted to the ICU and laboratory evaluation showed TSH < 0.01 mIU/L, FT4 5.00 ng/dL, FT3 9.9 pg/mL and urinary iodine 1080 µg/24 h.

Despite intense diuretic therapy and optimized thyrotoxicosis treatment (thiamazole 75 mg, dexamethasone 10 mg, cholestyramine 24 g, and lithium 800 mg daily), no significant improvement was observed, and since drug dosages and treatment duration were already reaching reasonable risk vs benefit, a multidisciplinary meeting – endocrinology, endocrine surgery, cardiology, and nephrology teams – was held to assess definitive therapeutic possibilities. Radioiodine therapy was ruled out due to his high urinary iodide and salvage thyroidectomy was declined in face of patient’s cardiac condition and severe thyrotoxicosis. Plasmapheresis, as a bridge to surgery, turned to be an impractical option, as the necessary volume overload would put the patient at an unacceptable risk.

**Figure 1:**
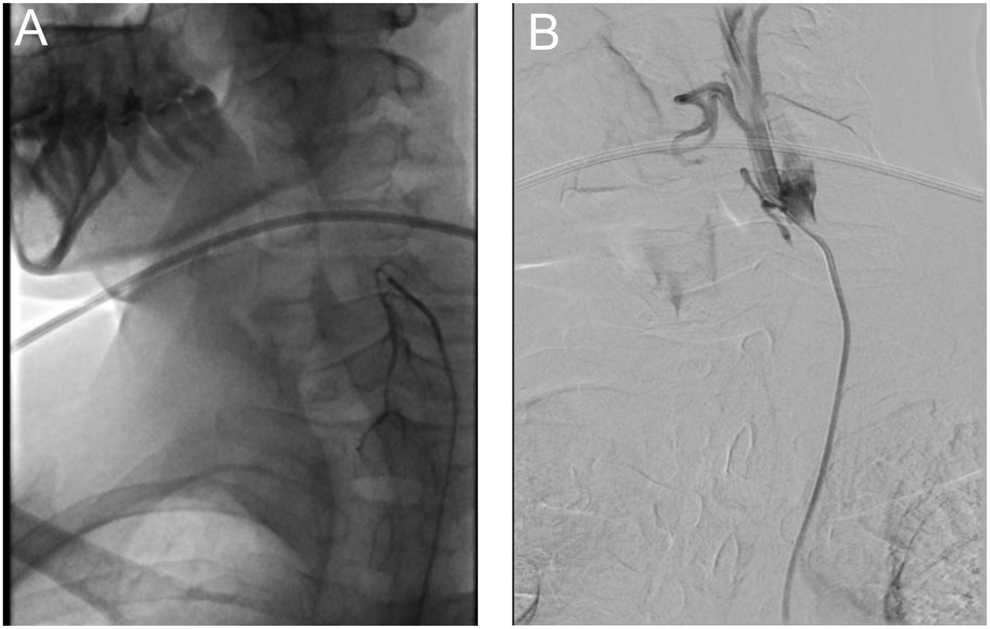
Angiography – Left superior thyroid artery before (A) and after (B) embolization.

**Figure 2:**
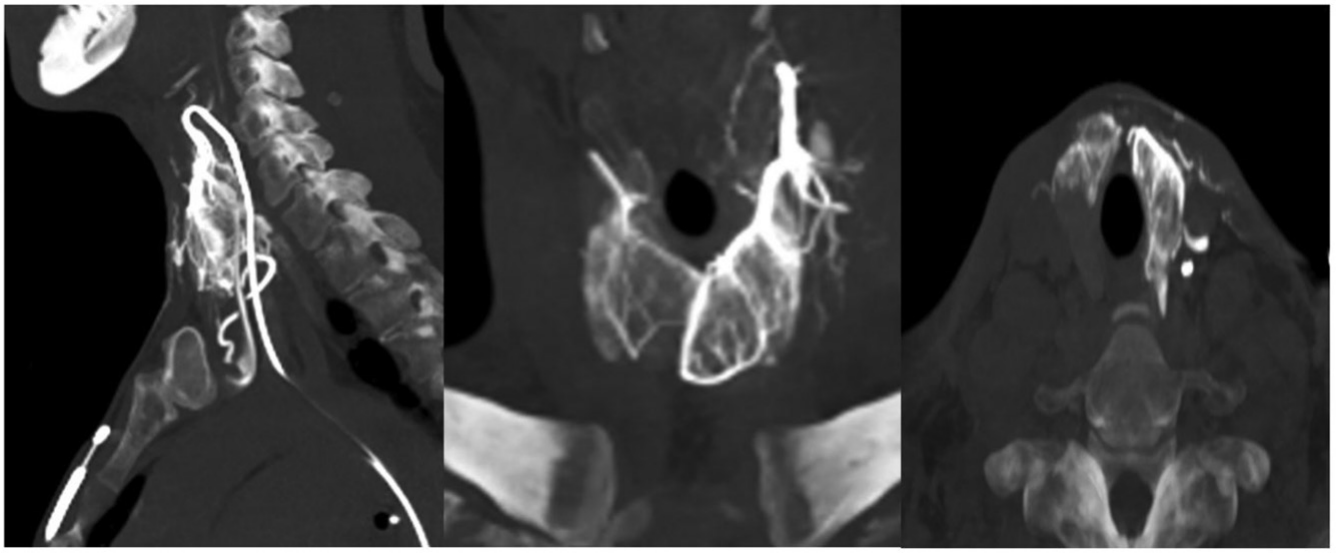
CT scan confirming left superior thyroid artery vascularization.

TAE of the four thyroid arteries was decided as a salvage therapy, which the patient accepted, after informed consent. The procedure was performed, on September 2019, by the interventional radiology team with polyvinyl alcohol 250–300 µm particles using an hybrid angiography and CT scanner (Nexaris, Siemens, Marburg, Germany) as shown in Figs 1 and 2. Both inferior arteries and then the superior arteries were successfully embolized, using a microcatheter 2.7F (Progreat, Terumo, Tokyo, Japan). The procedure took approximately 2 h and was done under local anaesthesia, with no immediate complications. Slight sore throat and burning sensation over the anterior neck were reported by the patient in the next few days, but without relief medication needed.

**Figure 3:**
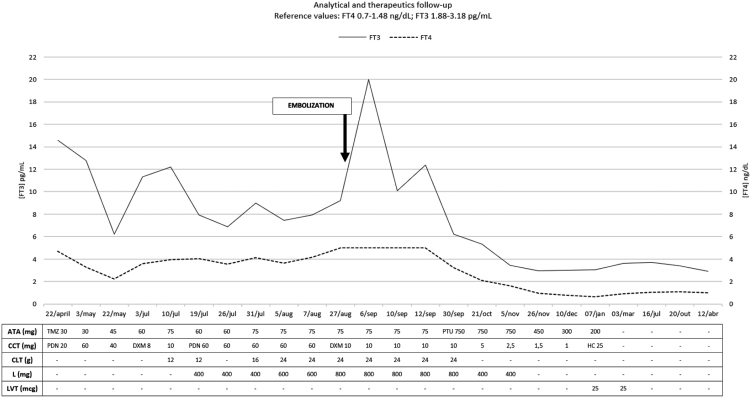
Analytical and therapeutics evolution. ATA, anti-thyroid agent; CCT, corticosteroid; TMZ, thiamazol; PTU, propylthiouracil; PDN, prednisolone; DXM, dexamethasone; HC, hydrocortisone; CLT, cholestyramine; L, lithium; LVT, levothyroxine.

On the 3rd day after TAE, blood tests showed TSH 0.01 mIU/L, FT4 5.0 ng/dL and FT3 20.0 pg/mL, and thiamazole was replaced by propylthiouracil. Analytical and clinical improvement were notably progressive and hence cholestyramine and lithium were stopped, dexamethasone was gradually reduced and switched to hydrocortisone on physiologic doses, and propylthiouracil was reduced accordingly (Fig. 3).

**Figure 4:**
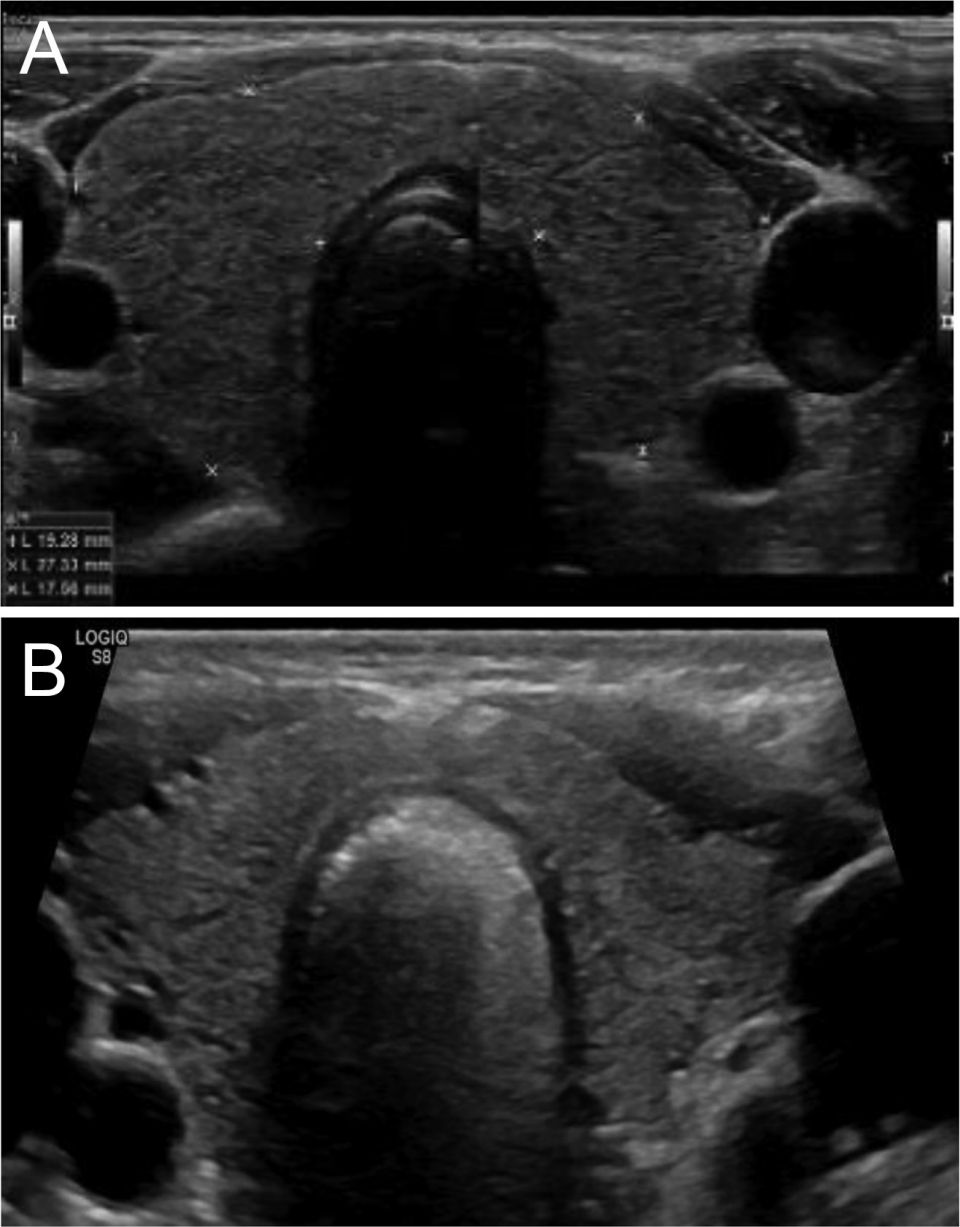
Thyroid ultrasound performed before (A) and 18 months after thyroid embolization (B) depicting a volume reduction of the right lobe of 44%, of the left lobe of 58%, and of the isthmus of 44%.

Four months after TAE, no tachycardia episodes were reported and the patient referred physical condition improvement with growing tolerance for exercise. At this point, he had cortisol 9.1 mg/dL, ACTH 22.7 pg/mL, TSH 25.2 mIU/L, FT4 0.63 ng/dL and FT3 3.06 pg/mL; hydrocortisone and propylthiouracil were stopped and levothyroxine 25 µg was introduced. Nine months after TAE, levothyroxine was suspended and thyroid function reevaluation showed TSH 2.82 mIU/L, FT4 1.04 ng/dL and FT3 3.69 pg/mL. At the last clinical observation, more than a year and a half after TAE, the patient was on bisoprolol 5 mg od, dofetilide 500 µg bid, bosentan 125 mg bid, furosemide 20 mg od and apixaban 2.5 mg bid, being able to carry out considerable physical effort as he was before the AIT episode. Laboratory evaluation showed TSH 3.5 mIU/L, FT4 1.0 ng/dL, FT3 2.9 pg/mL and urinary iodine 160 µg/dL (100–199). Thyroid ultrasound performed 18 months after embolization showed a homogeneous gland measuring right lobe 54 × 18 × 17 mm (volume of 9 cm^3^, volume reduction of 44%); left lobe 50 × 14 × 14 mm (volume of 4 cm^3^, volume reduction of 58%) (longitudinal/transverse/anteroposterior diameters); isthmus 5 mm (reduction of 44%), the diffusely hypoechoic, heterogeneous and hypovascular echogenicity of the thyroid gland was replaced with a standard hyperechoic echogenicity, with no signs of scaring or nodules (Fig. 4).

## Discussion

AIT is recognized as a diagnostic and management challenge, sometimes leading to life-threatening complications (5). The lipophilicity of the drug results in tissue storage for long periods, the reason why thyrotoxicosis may take several months to subside after amiodarone is suspended (8). The decision to maintain or discontinue amiodarone is a matter of debate in AIT. In our patient, the drug was stopped early during the course of the disease, according to the cardiologist’s advice. AIT is traditionally classified into two main types: type 1 AIT, characterized by an iodine-induced hyperthyroidism, less common; and type 2 AIT, as a result of destructive thyroiditis. Sometimes, mixed forms are recognized, where both pathogenic mechanisms are present. Thyroid scintigraphy was not performed, but the doppler ultrasound showed a hypovascular thyroid, characteristic of type 2 AIT (6). In addition, the time elapsed from the onset of amiodarone to development of thyrotoxicosis, the absence of goiter and TRAb are also characteristics of this type of AIT (6). However, increased thyroid dimensions on ultrasound, the persistent high levels of FT3, the resistance to treatment with high doses of glucocorticoids for such long period and the maintenance of euthyroidism even after cure, do not favor this hypothesis (6).

Due to the patient’s cardiac condition, thyrotoxicosis’ exuberance and urgency to ameliorate symptoms, a mixed type AIT was assumed and thionamide and glucocorticoids were started, as they are the mainstay therapy, aiming to address both forms of AIT (6).

As already mentioned, treatment’s refractoriness with worsening of patient’s clinical and analytical condition, despite the institution of high-dose glucocorticoids, forced an increase in thionamide’s dose in an attempt to stabilize thyroid hormones’ levels, even considering its potential toxic effects which remained monitored with complete hemogram and measurements of liver enzymes.

In cases of poor response or refractoriness to this therapy, adjunctive medical treatment is required, namely when a euthyroid state is urgent to achieve. Cholestyramine, by interfering with enterohepatic circulation and recycling of thyroid hormones, can reduce its levels (9) and lithium decreases thyroid hormone secretion (10). Hence, these two drugs were prescribed to our patient, but no clear efficacy was observed despite increasing dosages.

Potassium perchlorate by inhibiting thyroidal iodine uptake, may increase response to thionamides (11), but this was not an option owing to its unavailability. Sodium perchlorate, as an alternative, was also unavailable.

The refractoriness of thyrotoxicosis after several months of multimodal medical therapy and the patient poor cardiac condition demanded other approach to achieve euthyroidism. Thyroidectomy was then considered as the European Thyroid Association recommends (6).

Studies concerning thyroidectomy as treatment for AIT patients are lacking, data on survival for these patients are heterogeneous, and no recognized factors could identify patients who might benefit from it. Recently, Cappellani *et al.* compared outcomes on survival of total thyroidectomy vs medical therapy in AIT patients, demonstrating that overall mortality and cardiac-specific mortality were lower in the surgery group, even before euthyroidism was restored (12).

Given our patient’s cardiac condition and hyperthyroid state, performing thyroidectomy would require intraoperative monitoring of his hemodynamic status to preclude the dreadful possibility of a thyroid storm through invasive blood pressure monitoring and echocardiographic imaging of the heart. Furthermore, the risk of associated dysrhythmias caused by release of catecholamines and even thyroid hormones due to gland manipulation was a major concern. In order to prevent these complications, these physiologic responses would have to be blunted with further medication. The mandatory need to avoid any further increase in pulmonary arterial pressure, excessive adrenergic stimulation and maintain systemic vascular resistance would turn to be a major challenge while performing thyroidectomy in our patient.

After considering these cumbersome facts, the medical team decided to purpose thyroidectomy as definitive treatment, but only after stabilization of thyroid hormone levels to near normal limits, which would be possible after apheresis, in order to minimize some of the described risks above. Plasmapheresis can be used in patients with severe thyrotoxicosis when first-line therapies fail or cannot be used, leading to a decrease in the hormone concentrations. This effect is transient and hormone levels typically rise again in the next few days (13). When considering apheresis, some complex issues have arisen in this case. On one hand, the procedure would imply volume overload, a further increase in pulmonary arterial pressure (already at 120 mmHg) and excessive adrenergic stimulation, which the patient would not be able to tolerate (14). On the other hand, the necessary fluids would dilute blood hemoglobin (in this patient around 21.0 g/dL) which would compromise oxygenation (13). At this point, plasmapheresis turned to be an impractical option, and so was thyroidectomy.

TAE appeared as a salvage therapy in this patient. It has been used for critically ill patients whose condition is refractory to medical therapy and too unstable to undergo thyroidectomy (15, 16). However, TAE is a procedure without established protocols and has been used in a few cases of thyroid disease, namely large goiters, Graves’ disease, and thyroid carcinoma, although rarely reported in AIT, with no published controlled trials (17, 18). Most authors describe its effects as similar to subtotal thyroidectomy, suggesting that the number of embolized arteries should depend on arterial supply evaluated by previous angiography (15, 19). Main facts about previously reported TAE for the treatment of thyrotoxicosis are summarized in Table 1.

**Table 1:** Previously reported thyroid artery embolization for treatment of thyrotoxicosis.

Authors	Disease	Number of patients	Number of embolized arteries	Arteries embolized	Procedure length (hours)	Surgery	Complications (n)	Outcomes
Dedecjus *et al.* (17)	TG	10	3	2 superior + 1 inferior	<1	Thyrodectomy in the next 36 h	Hematoma 2 Fever 2 Neck pain 2	Shorter operation time, reduced drainage, and blood loss
Xiao *et al.* (15)	GD	22	2 in 17 3 in 5	All 2 superior + 1 inferior in 5	<1.5	Thyroidectomy in 6 2 weeks after	Neck pain 22 Fever 19 Transient hypocalcemia 1	Thionamide maintenance 2
Zhao *et al.* (16)	GD	28	3 in 22 2 in 6	NI	NI	NI	Neck pain 28 Transient hypocalcemia 1 Transient hypothyroidism 1 Fever 1	Euthyroidism 22 Reembolization 2 Thionamide maintenance 5 Recurrence 1 Thyroid gland size decreased in all
Brzozowski *et al.* (18)	GD TG AIT	5 8 2	2 in 67% 3 in 33%	All inferior + 1 superior	NI	NI	None	Volume reduction Thionamid maintenance 7 Improved orbithopaty
Rohr *et al.* (20)	AIT	1	4	All	NI	Thyroidectomy 11 days after	None	Cured
Kaminski *et al.* (7)	TG GD AIT Goitre	8 7 2 5	Most 3	NI	2	NI	Neck pain 22 Fever 12 Transient hypocalcemia 5	Euthyroidism or Hypothyroidism 70.6% Volume reduction
Zhao *et al.* (16)	GD	1	4	NI	NI	NI	None	Cured
Tartaglia *et al.* (21)	TG	1	3	1 superior + 2 inferior	NI	NI	Neck pain	Volume reduction by half Resection through the cervical approach alone
Tartaglia *et al.* (22)	HT	10	All in 5 3 in 5	NI	NI	Thyroidectomy in 3 30, 30, and 60 days after	None 8 Hemilarynx palsy 1 Laryngeal nerve palsy 1	

GD, Graves’ disease; HT, hyperthyroidism; NI, no information; TG, toxic goiter.

In this patient, due to the emergent need to control thyroid function, we decided to perform TAE of the four thyroid arteries. To the best of our knowledge, few case reports in the literature have described the embolization of the four thyroid arteries in AIT context (16, 20).

The rise in thyroid hormone levels on the first week after the procedure was expected, owing to the release of thyroid hormones due to ischemia and necrosis of embolized thyroid tissue. Although TAE reduces thyroid blood supply, the veins remain unconstrained and colloid from dying thyrocytes (comprising thyroglobulin, T3, T4 and, probably, other biochemical compounds) gets into circulation (17). Although the rise in thyroid hormone concentration was related to the destructive process due to the procedure, thiamazole was replaced by propylthiouracil as the latter has inhibitory effect on T4 conversion to T3. Despite this, no worsening of symptoms of hyperthyroidism occurred. Neck pain referred by the patient was reported in nearly 85% of the cases (18). Parathyroid glands are believed to be supplied by blood vessels originating from inferior thyroid arteries. As a prevention of potential parathyroid function harm, it has been recommended not to embolize both of them (7). Concerning this fact, calcium homeostasis was evaluated immediately after the procedure and in the subsequent blood analysis, but no significant changes in serum calcium levels were observed.

Given the four thyroid arteries embolization, permanent hypothyroidism could be expected. However, it turned out to be transitory as the patient stayed euthyroid after levothyroxine withdrawal. The authors believe that incomplete ischemic destruction and/or neovascularization processes or partial recanalization of embolized arteries might be the explanation (15).

In conclusion, TAE proved to be the only effective treatment in this patient. Endovascular embolization techniques are a valuable therapeutic option and can be considered in cases where standard forms of treatment are ineffective or involve unacceptable risks.

## Declaration of interest

The authors declare that there is no conflict of interest that could be perceived as prejudicing the impartiality of this case report.

## Funding

This work did not receive any specific grant from any funding agency in the public, commercial, or not-for-profit sector.

## Statement of ethics

Patient has given his written informed consent to publish his case (including publication of images).
